# Balance Performance across the Lifespan Assessed by the Leonardo Mechanograph^®^: A Cross-Sectional Study

**DOI:** 10.3390/jfmk5010001

**Published:** 2019-12-19

**Authors:** Sabine Wiegmann, Dieter Felsenberg, Ulf Gast, Hendrikje Börst, Gabriele Armbrecht, Roswitha Dietzel

**Affiliations:** 1 Charité–Universitätsmedizin Berlin, corporate member of Freie Universität Berlin, Humboldt-Universität zu Berlin, and Berlin Institute of Health, Centre for Muscle and Bone Research, Hindenburgdamm 30, 12200 Berlin, Germany; 2 Centre for Muscle and Bone Research, Charité-Universitätsmedizin Berlin, 12200 Berlin, Germany; dieter.felsenberg@charite.de (D.F.); tendaba@gmx.net (U.G.); gabi.armbrecht@charite.de (G.A.); roswitha.dietzel@charite.de (R.D.)

**Keywords:** postural control, normative values, force plate, center of pressure, COP, balance, Leonardo Mechanograph, posturography

## Abstract

Reference values of sway parameters have not been published for the Leonardo mechanograph^®^ so far. The aim of this cross-sectional study was to determine normative values on postural control measured by the force plate Leonardo Mechanograph^®^ and to analyze the influence of age and sex on balance performance. A set of standardized standing positions with eyes opened (Romberg, semi-tandem, tandem, unipedal standing) was carried out. Analysis of covariance (ANCOVA) was used to detect age-and sex-related differences in center of pressure (COP) parameters (path length, velocity, elliptical area, anterior-posterior, and medio-lateral directions). Measurements were available for 570 subjects aged 20–86 years. Statistical analysis showed a high effect of age group on postural control (partial n^2^ between 0.1 and 0.4) with a U-shaped dependency between postural control and age for all area- and path-related COP parameters, with the largest sway in the youngest (aged 20–40) and the oldest age group (aged 60–86). For velocity of COP, a linear deterioration with increasing age was found. Medio-lateral components of COP are likely to indicate the extent of postural control. Significant sex differences were not clearly supported by current findings. Age- and sex-related normative values are a useful resource for diagnostic, research, and training.

## 1. Introduction

The Leonardo Mechanograph^®^ is a commonly used system for posturography in clinical environments as it is easily used by health care practitioners. It is recommended as an innovative, inexpensive, and precise way to assess motor performance not only in research environments but also in the fields of physiotherapy, sports, or medical diagnostics [1]. While normative values do exist for other force platforms [2,3] and assessments of postural control [4,5], no normative values of sway parameters have been published for the Leonardo system as of yet. Normative values can help healthcare practitioners in a clinical, research, or training setting to assess the performance of a person compared to people of the same age group. The purpose of this cross-sectional study is to report on normative values on postural control measured by the Leonardo Mechanograph^®^ force plate and to offer some insight into the relationship between age, sex, and postural control for a differentiated view on earlier investigations.

Postural control is a fundamental motor skill that can be defined as “the act of maintaining, achieving or restoring a state of balance during any posture or activity” of daily life [6]. A body is in balance when the line of gravity falls in the base of support. When the line of gravity moves out of the base of support, the body becomes unbalanced, increasing the danger of falling. The interaction of the visual, vestibular, and somatosensory systems activates muscles and balancing mechanisms to prevent falling. The ankle, hip, and stepping strategies are the ones most commonly described [7,8]. Like any other motor skill, postural control improves during childhood and adolescence. Some authors describe a U-shaped dependency between balance and age, with the least amount of postural control in early childhood and old age. Maximum postural control is achieved between the ages of 20 and 60 years [3,9,10]. Like any other motor skill, postural control strategies depend on an individual’s physical activity and can be improved via training [7,11].

The loss of postural control from early adulthood to old age is a natural development in which several factors play a role. Due to the reduction of cognitive functions and a deterioration of the sensorimotor system, postural control in older people becomes increasingly restricted. Elderly or physically inactive people have less sensory input, and the responses of their neuromusculoskeletal system are reduced. This is associated with a decline in muscle strength, decreased knee or plantar reflexes, and reaction times—thus, a reduction in effective protective movements [12]. Poor balance control is mostly associated with an increase in the postural sway, which correlates with fall risk [13,14].

Computerized force platforms measure the displacement of the center of pressure (COP). The COP is calculated from the ground reaction forces of the body and is described as the location of the vertical reaction vector on the surface of a force platform. COP movement reflects the responses of the muscles and the balance mechanisms that work to keep the center of gravity over the base of support to resist disturbances and thereby falling. These COP movements are widely known as postural sway [15].

Researchers have used various methods in assessing balance. The most common procedures are based on the Romberg tests, applied during various standing conditions (e.g., tandem or one leg stance) and with eyes opened or closed [14]. The degree of postural sway is generally measured using static or dynamic posturography [16].

Technical literature describes various parameters of COP movements to characterize postural sway. The most commonly documented outcomes are mean velocity, mean distance/path length, mean frequency, sway area, anterior-posterior (AP) and medio-lateral (ML) displacement of the COP. According to a systematic review of Piirtola and Era [17], the most predictive outcomes for balance disorders are the mean or root-mean-square of ML COP movement, ML amplitude, or mean speed of COP movement.

The primary purpose of this study is to report on a normative dataset of postural control in a random sample of German subjects aged 20–86 years. An additional aim is to analyze the influence of age and sex on various sway parameters of postural control.

## 2. Materials and Methods

### 2.1. Participants

The present study was part of the German “muscle survey” project, a population-based, cross-sectional investigation examining muscle and bone health. This article focuses on the investigation of postural control. A random sample of participants was taken from the resident registration office in Berlin, Germany. As a first step, the potential participants were screened for suitability. As the study also focused on norm data for body composition using dual X-ray-absorptiometry, subjects were excluded who (1) had metal implants or artificial prostheses; (2) had edema; (3) took medication affecting water-mineral homeostasis; (4) needed a walking aid; (5) had contraindications for X-ray exposure; (6) were pregnant; (7) were unable to provide informed consent or to follow the instructions. Those who met the inclusion criteria gave their written informed consent to participate in this study. The study was conducted in accordance with the Declaration of Helsinki, and ethical approval was granted from the ethics committee of Charité-Universitätsmedizin Berlin (EA4/095/05).

**Figure 1:**
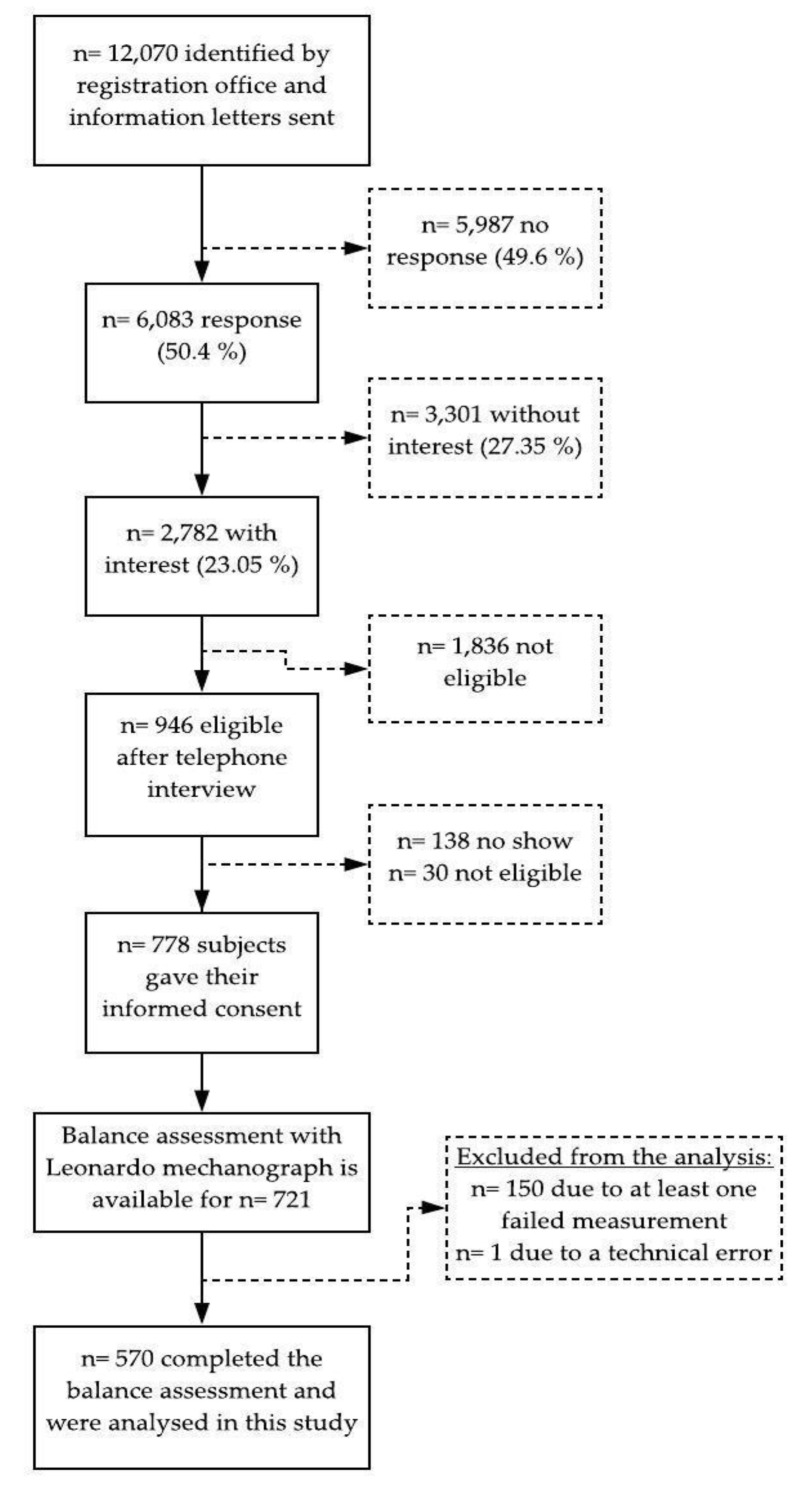
Flowchart of the study.

Of a total of 721 participants, 570 subjects were able to complete the balance assessment with their eyes open and thus included in the analysis. The missing values resulted from subjects losing their balance during the tests and one data set was incomplete due to a technical error. An overview is given in a flowchart of the study (Figure 1).

### 2.2. Measures

#### 2.2.1. Anthropometry

Body weight and body height were determined to the nearest 0.1 kg and 0.1 cm, using an electronic measuring and weighing station (Seca 764). All participants were measured between 09:00 and 11:00 in underclothing and barefoot.

#### 2.2.2. Posturography

Balance Mechanography was performed with the Leonardo Mechanograph^®^ Ground Reaction Force Plate (Novotec Medical GmbH, Pforzheim, Germany software package 4.2). All measurements were sampled at a frequency of 800 Hz. For the assessment of balance parameters, a low-pass filtering with a FIR filter with 30 sampling points (30 taps) and a cut-off frequency of 8 Hz was used.

### 2.3. Design and Procedures

The balance tests were structured hierarchically according to degree of difficulty. Before the measurement, the examiner demonstrated all assessment positions to the participants. There were no practice trials. Each test should be conducted once for 10 s. The subject should stand as still as possible for approx. 2 s, otherwise the measurement will not start. The measurement was started by the examiner pressing the START button. A single beep indicated the start of the measurement, a double beep after 10 s indicated the end. The software automatically counted the seconds backwards from 10, which was shown in large letters on the display. The tests were done with eyes opened. If the participant was unable to perform the test, that is, if they had to take a step or hold onto the wall during the test, the test was halted, and no further balance tests were performed. For safety reasons, the examiner stood diagonally in front of the test person in order to be able to catch or provide support if necessary.

This study considered the standard test conditions and circumstances of the International Society of Posture and Gait Research, such as the arrangement of the room, the fixation point of the eyes, the position of the feet, the influence of the visual system or interindividual differences [18]. The motion laboratory was equipped with a Leonardo Mechanograph^®^, a desk for the computing device, a chair for resting, and an examination table. The force plate was connected to the laptop via a USB cable and stood outside the area of movement of the force plate. The device was installed on a solid and even surface and 1 m away from every wall in the laboratory. The force plate was adjusted so that all six posts of the plate touched the floor equally and no rattling sound could be observed. The room had normal illumination and was large enough to avoid acoustic spatial orientation. Only the examiner and the participant were in the motion laboratory, so that the measurement was not influenced by confounding factors such as sound or vibration sources [18].

During each test, the participant had to fix on a red cross placed at eye level about 4 m away. In order to reflect real-life conditions, they wore their own comfortable flat shoes and clothes. They had to stand as still as possible, in an upright position with both arms hanging relaxed on the force platform. They could use their arms to keep balance.

The first position was the Romberg stance, in which the participants had to stand with their feet close, touching each other. The assessment continued with the second position, semi-tandem standing. Starting from the Romberg position, one half-foot step was taken, the heel of one foot aligned with the toe of the other. After that, the third position, tandem standing, was performed. The subject put both feet in a line, with the heel of the front foot touching the tip of the back foot. For the semi-tandem and tandem stances, the participant could choose which foot was standing in the front and in the back. The fourth position was the one leg stance. First, the participant had to stand on the right leg, with the left leg raised slightly, without touching the other leg. The same procedure was carried out with the left leg.

Relevant outcome parameters provided by the Leonardo software such as path length, the area of sway, the mean velocity of COP, and the velocity and path length in the anterior-posterior (AP) and medio-lateral (MP) directions were included in the statistical analyses [17] (Table 1).

### 2.4. Statistical Analysis

Statistical analyses were carried out using SPSS version 25, and the open source statistical software R version 3.6.1, to perform scatterplots and bootstrapping (The R Project for Statistical Computing, www.r-project.org). Descriptive statistics were generated by characterizing the sample, anthropometric data, the balance-performance variables, and the frequency of test performance. In testing for normal distribution, both a Kolmogorov–Smirnov test and a graphic interpretation of the histogram and the Q-Q-plot were carried out. Due to nonparametric distribution, the COP parameters are presented in median, 1st and 3rd quartiles. All other data are described as mean and standard deviations or given in percentages. An independent t-test was carried out to compare the persons who were excluded from the analysis because they failed at least one test (*n* = 151) with those who completed all tests (*n* = 570).

To get a normal distribution of the balance variables, a logarithmic transformation was applied to all of them. To explore the relationship between age and the COP parameters, scatterplots as well as bivariate and partial correlations were performed.

After verifying all requirements, analysis of covariance (ANCOVA) was finally carried out to analyze the influence of age and sex on balance performance after controlling for the covariates of body height and weight. Pairwise comparisons were performed to identify differences in age groups and sex. A Bonferroni correction was applied to correct for multiple testing. The statistics of the R^2^ and adjusted R^2^ were evaluated using the bootstrap resampling (*n* = 10,000). The alpha level error was set at *p* < 0.05 for all analyses.

### 2.5. Age

This study examines the relationship between age and balance performance. Thus, age was recorded in years and categorized into three age groups for the analysis. This transformation helped to deal with nonlinearity and to present the results in a more clear and practicable way.

The youngest age group comprised individuals aged between 20 and 40 years and is referred to as ‘young adults’. Balance control has reached an optimum and motor performance is the most advanced. Subjects between 41 and 60 years were grouped into ‘middle-aged adults’. Depending on the level of physical activity, this age may already show a slight decline of postural control and motor performance. Individuals aged 61 and older represent the ‘old adults’. Their motor development can be defined by a pronounced reduction of physical performance [10,19,20].

## 3. Results

The study group consists of 289 (50.7%) women and 281 (49.3%) men. The mean age was 48.5 years (SD 16.7). Mean values of subject height and weight were 171 cm (SD 9.45) and 76.4 kg (SD 14.08). Table 2 presents the descriptive statistics of the study population.

**Figure 2:**
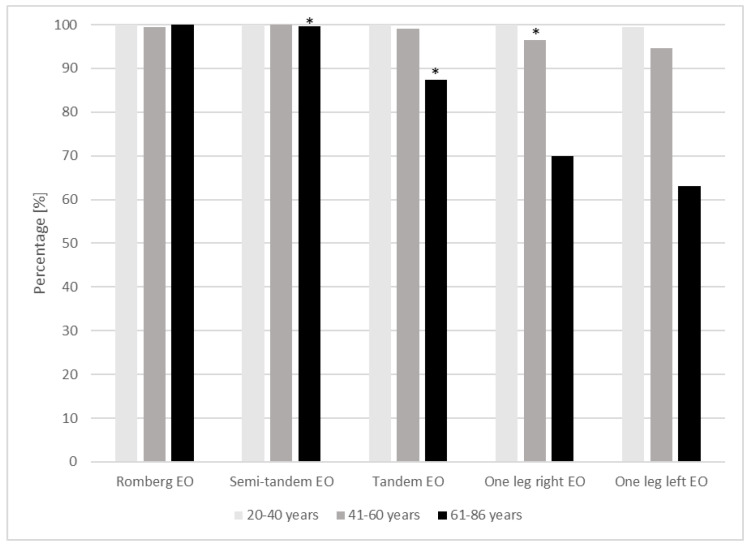
Age-group related overview of the ability to perform the balance tests with eyes open (EO) (*n* = 721), * significant difference with *p* < 0.05 for at least one COP parameter between the groups “test failed” (*n* = 151), and “test completed” (*n* = 540) evaluated by independent *t*-test by age group.

Figure 2 shows an age-group related overview of the ability to perform the balance tests and illustrates the reduction in the sample size due to failed tests. Nearly all subjects were able to maintain balance in the Romberg and semi-tandem positions. In the more demanding test positions (reduced base of support), the differences between the age groups became clearer, e.g., the older the participants, the higher the percentage of failure (Figure 2). No obvious difference in the failure rate of men and women could be observed. A significant difference between those who failed at least one test and those who completed all tests could be observed for the oldest age group in the semi-tandem and tandem stances, as well as for the 41–60 year age group in the one leg stance on the right leg.

### 3.1. Age-Related Differences

Accounting for the effects of age, each COP parameter correlates significantly with each other. There is always a moderate to strong correlation (Table S1).

Supplementary Table S2 shows the descriptive statistics of all COP parameters for each five-year band between 20 and 86 years classified by sex. This table as well as the scatterplots in the Supplementary Figures S1–S4 can serve as a reference tool for practitioners.

Supplementary Table S3 shows the results of the ANCOVA and pairwise comparisons presented by adjusted means, standard error (SE), and 95% confidence interval of all COP parameters in all standing positions. To get untransformed values, the values must be converted like this: e^mean^.

The means show that the oldest age group has the highest values. Differences can be seen between the youngest and middle-aged groups. In all standing conditions, the 41–60 year olds achieve lower means for PLen, PLenX, and PLenY; also in both single leg stances for StdElA. For all variables related to the velocity of COP, the youngest age group has the lowest means (Table S3).

It can be observed that within an age group, by reducing the base of support, the means increase. It is noticeable that the means for the area-related measurement (StdElA) for both one leg stances increases threefold in all age groups (Table S3).

Medio-lateral components of the path length and speed of COP (PLenX or BtVmeanX) are mostly more pronounced than the anterior-posterior displacement of COP (PLenY or BtVmeanY). The smaller the base of support became, the more pronounced was the difference between medio-lateral and anterior-posterior displacement (Table S3).

ANCOVA revealed a statistically significant effect of age group on all dependent variables after controlling for body weight and height (*F*-value with *p* < 0.001 for all variables). The effect of age group on postural control can be classified as high (partial η^2^ between 0.1 and 0.4) (Table S3). Pairwise comparisons always showed a significant difference between the middle and old age groups (*p* < 0.001) and the youngest and oldest age groups (*p* < 0.001). Comparing the estimated marginal means showed that the largest COP oscillations were in the oldest age group compared to both of the other age groups (Table S3). However, there is not always a highly significant difference between the youngest and middle-aged groups (Table S3). The 41–60 year olds often achieve better results than the youngest age group, especially for path-related (PLen, PLenX, PLenY) and area-related parameters (StdElA). Regarding the speed-related parameters (BtMeanvCoF, BtVmeanX, BtVmeanY), a continuous increase in the balance results from young over middle to old were identified.

Table 3 shows the ANCOVA summary models with the bootstrapped 95% confidence intervals for each standing position: for the Romberg, the model with the variable PLenY provides the best explanation of the variance with F (7, 562) = 53.934, *p* < 0.001, adj. R^2^ = 0.39 (0.34; 0.47). For the semi-tandem, the model with the variable PLen provides the best explanation of the variance F (7, 562) = 61.737, *p* < 0.001, adj. R^2^ = 0.43 (0.36; 0.51). For the tandem, the model with the variable PLenX provides the best explanation of the variance F (7, 562) = 52.879, *p* < 0.001, adj. R^2^ = 0.39 (0.33; 0.46). For both one leg stances, the variable StdElA provides the best explanation of the variance F_right_ (7, 562) = 78.202, *p* < 0.001, adj. R^2^ = 0.49 (0.43; 0.56) and F_left_ (7, 562) = 66.83 *p* < 0.001, adj. R^2^ = 0.45 (0.39; 0.52) (Table 3).

### 3.2. Sex-Related Differences

The effect of sex on postural control must be classified as small, because partial η^2^ is always less than 0.02 (Table S3). Only in a few variables could a statistically significant influence be found, especially during the one leg stand on the right side. This was particularly clear for the variables that measured the anterior-posterior displacement of COP (PLenY and BtVmeanY). They achieved a significant *F*-value in all standing conditions (*p* = 0.001–0.03). It was demonstrated that the anterior-posterior displacement in women is significantly lower than in men. In general, men tend to achieve worse results than women in all positions and variables. Only in the middle-aged group were men better than women, especially during tandem and unipedal standing, but not significantly so (Table S3).

## 4. Discussion

In this German cross-sectional study, data on the postural control of men and women between 20 and 86 years of age were studied. The aims were to obtain normative data for various COP parameters using the Leonardo Mechanograph^®^ and to analyze the influence of age and sex. The main findings supported a strong relationship between age and postural control. It was shown that there is a U-shaped dependency between postural control and age for path- and area-related COP parameters, respectively, and a linear dependency for speed-related COP parameters. The medio-lateral sway played, apparently, an important role in predicting balance ability. It would seem that women have better postural control than men.

### 4.1. Age-Related Differences

Several authors discussed a U-shaped relationship between age and postural control with the best results in the ages between 20 and 60 years [3,9]. The current findings give a differentiated view of the U-shaped development, as it does not apply to all COP parameters. The U-shaped development refers to the path-related parameters (PLen, PLenX, PLenY) in all standing conditions and area-related parameters (StdElA) in the one leg stances. This result is in line with a recent study by Goble and Baweja [2]. They observed the U-shape for the path length of COP with the first balance decline in the 40–49-year old age group. In contrast, the current data showed a linear deterioration for speed-related parameters. This result contradicts that of Era et al. [3] and Hytönen et al. [9], who tested different COP velocity parameters and discovered this U-shaped development in all of them. The question arises why speed-related parameters provide a different picture in this study. All these studies have used different force plates with frequency ranges between 50 and 1000 Hz. In particular, regarding the measurement of speed, this can make a significant difference. The test duration varied between 10 s in this study and, respectively 20 s [2], 30 s [3], or 3 min [9] in the other studies, which might produce different results. Furthermore, comparability with other studies is difficult, as all used different sample sizes, age group classifications, or statistical methods [21]. The current standardization protocol for posturography was published by the International Society of Posturography in 1983 and needs to be updated [18]. Due to the absence of a valid recommendation on standardization, generalizing of the findings is restricted and requires further investigation.

In addition, the medio-lateral components of COP seem to be an important parameter to describe the extent of postural control. Data showed that the medio-lateral components of path length and speed (PLenX, BtVmeanX) are notably higher than the anterior-posterior components (PLenY, BtVmeanY) for all ages and test conditions. This becomes particularly clear in the demanding test conditions and in the oldest age group. Similar findings have been found in Morrison et al. [22], who compared postural control of patients with multiple sclerosis (MS) and healthy persons. They observed that MS patients, who obviously have reduced postural control, have higher medio-lateral sway than healthy people. They summarize that the more difficult a balance position is, or if the balance ability is reduced, the more the hip strategy is used. Therefore, the medio-lateral sway becomes larger. Pasma et al. [23] conclude in their study that COP displacements in the medio-lateral direction could be useful parameters to evaluate age-related differences in quality of standing balance. There are also findings that used medio-lateral sway to distinguish between fallers and non-fallers, especially in narrow standing conditions, e.g., tandem standing [17,24].

Further evidence suggests that there is a relationship between narrow foot positions and medio-lateral sway. In narrow stances with the feet kept together, in contrast to freely chosen and comfortable feet positions (like in normal standing), higher sway in the medio-lateral direction was found [25]. In this study, a standardization of foot position for Romberg, semi-tandem, and tandem standing was used. This required an increased postural strategy because the support surface decreased progressively. In consequence, the medio-lateral components increased. During unipedal balance tasks, the medio-lateral displacement was the highest. That supports the assumption that challenging balance conditions as well as the feet distance produces greater COP movement in the medio-lateral direction [25,26,27].

### 4.2. Sex-Related Differences

The effect of sex on postural control must be considered marginal with a tendency to better balance in women, which is in line with results of earlier research [2,3,28,29,30]. Men had predominantly higher COP displacements than women. Earlier investigations have been inconsistent regarding the differences between males and females. There were also studies that have reported better results in males [31] or no significant differences between the sexes [25]. One consideration would be whether the lack of standardization and inconsistent use of measuring instruments, procedures, and analyses has led to different results. This assumption cannot be confirmed by comparison with the studies that achieved similar results to those provided by this study. Despite different measuring instruments (Good Balance force plate [3], AMTI [29], Kistler [28]), statistical analysis (linear regression [3] vs. ANCOVA [28]), and test duration (20–30 s [3] vs. 40 s [28]), the authors came to the same conclusion that women achieve better results than men. In the study of Overstall et al. [31], in which men performed better than women, the test duration was the longest at 1 min. It is possible that the balance performance of men and women changes over time, which needs further investigation.

Another possible bias may have occurred due to the standardization of the foot position in this analysis. It is known that taller subjects prefer a wider distance between their feet to stand in a stable manner [32]. In addition, the length of the feet can influence the postural control but was not evaluated in this study [30]. Other influencing factors discussed in the literature, such as a sex-related difference in the tactile perception of the feet in relation to body size, can also explain the poorer performance, especially in men [28].

The sex differences in the anterior-posterior direction (PLenY, BtVmeanY) can be explained by the different use of balance strategies. Women apparently have a better balance ability than men, and thus make more use of the ankle strategy. Persons with reduced postural control prefer the hip strategy [22].

### 4.3. Limitations

Finally, several potential limitations need to be addressed. First, this study did not consider the activity level of the participants. It can be assumed that the study group probably consists of high performing persons who were able to complete all balance tests with their eyes open. Different investigations support a relationship between highly functioning people and better balance performances [11,33]. In addition, the data analysis only included complete cases. Therefore, the sample size was reduced, which may have led to a bias of the results and reduced statistical power.

The high failure rate could be caused by the balance assessment itself. The increasing difficulty of the balance tests with a reduced base of support presented a challenge to the postural control system. The challenge rose with age and was even more pronounced from the age of 60. The effect was clearly obvious in the tandem and both unipedal standings. There is conflicting evidence whether unipedal stances are suitable assessment tests. Medell and Alexander [34] discuss the unipedal stance as a predictor for falls, because it strongly correlates with the time for stepping balance responses. Due to the large variance in rate of success, Speers et al. [35] recommend choosing tests of moderate difficulty such as Romberg, semi-tandem or tandem standing for clinical assessment, as the high failure rates during one leg standing indicate a too high degree of difficulty. Additionally, Bryant et al. [25] discuss whether the single leg stance is useful for testing balance ability, since over half of their study group failed to complete this trial. In contrast, Ponce-Gonzalez et al. [36] identified the single leg stance as a reliable assessment test for balance carried out in a static position with eyes opened, using the best result of six trials as reference. However, this study had a much younger and more physically active sample (mean age 23), which might have influenced the performance. At least the assessment of the unipedal stance offers the advantage of evaluating stability in a situation where the postural control system is challenged to the maximum. In consequence, further research should take age and activity levels into account when selecting the test for postural control. To avoid high failure rates in future research projects or in balance assessments, Romberg, semi-tandem, or tandem standing for assessment of heterogeneous cohorts are recommended. For the assessment of young people, athletes, or the active elderly who need a challenge for their balance system, unipedal standing is suitable.

A further limitation of this study may be caused by the classification into age groups. Despite the fact that the allocation to age groups was theoretically justified based on physiological motor development over the course of life [10,19,20], it did not take into account the apparently U-shaped development in postural control with a similar performance in the ages of 20–60 year olds [3,9]. The current study found highly significant group differences, but this was most visible between the youngest and oldest age groups as well as between the middle-aged and oldest age groups. The group difference between the first and second group was not clearly observable in all variables. This can be related to sampling and age group classification. This may have caused bias, particularly in the distinction between the young and the middle-aged group because the balance ability in these two age groups is probably similar, which would support the theory of U-shaped development with a performance peak between 20 and 60 years. Future research should use either a division into age decades or a classification that distinguishes between children (<18 years), adults (18–60), and the elderly (>60). This is likely to better reflect the U-shaped development. Additionally, caution is required when a statistical transformation is performed from a metric scale (age) to an interval scale (age groups), which can lead to information loss [37].

Furthermore, one must exercise caution due to the extensive analyses of many variables. Multiple testing can be accompanied by an increase in the alpha error. Therefore, Bonferroni correction was applied, but the values of F and *p* should be interpreted with care.

## 5. Conclusions

Despite its exploratory nature, this study offers some insight into the relationship between age and postural control and gives a differentiated view on earlier investigations. This study is the first to report normative values on postural control measured by the Leonardo Mechanograph^®^.

The Leonardo Mechanograph^®^ proved to be a practical tool to test the balance over the range of ages in different target groups. The normative values are a useful resource for research and practice. The short duration of the tests and the design of the examination environment (e.g., own shoes) make it possible to use them in the daily routine of physiotherapists, sports therapists, and physicians. For various diseases with balance disorders, only a test of a short duration is possible. For safety and time reasons, shoes often stay on. Therefore, the normative values serve as a simple reference system to assess whether the balance ability is within the norm. Based on the current values, the Leonardo software could be extended by a balance index, which indicates a performance value in relation to the age group reference. Prospective studies are needed to determine cut-off values for fall risk assessment.

The following conclusions can be drawn from the present study: this study supports the U-shaped development of postural control over age in the context of various COP parameters with the best performance in the middle-aged adults. The analysis revealed a linear increase from young to old in speed-related COP parameters, which needs to be analyzed in more detail. Medio-lateral sway seems to be an important factor to describe balance ability and to differentiate the used balance strategy. Women tend to have better postural control than men. The impact of physical activity should be investigated in further studies.

## Figures and Tables

**Table 1:** Description and abbreviations of the center of pressure (COP) parameters.

Variable	Abbreviation	Description
**Path-Related COP Parameters**		
Path length of COP	PLen	Total path length of the COP during the measurement, in mm
Medio-lateral component of the path length of COP	PLenX	Total path length of the COP in the medio-lateral direction, in mm
Anterior-posterior component of the path length of COP	PLenY	Total path length of the COP in the anterior-posterior direction, in mm
**Area-Related COP Parameters**		
Area of sway	StdElA	Standard ellipse area including 90% of all COP points during the measurement, in cm^2^
**Speed-Related COP Parameters**		
Mean velocity of COP	BtMeanvCoF	Mean speed of the movement of the COP over the time of the test path length/duration, in cm/s
Mean velocity of ML	BtVmeanX	Average speed of COP movement in the medio-lateral direction, in mm/s
Mean velocity of AP	BtVmeanY	Average speed of COP movement in the anterior-posterior direction, in mm/s

**Table 2:** Descriptive statistics of the study population (*n* = 570).

	Age Group			
Variable	20–40 yrs.	41–60 yrs.	61–86 yrs.	Total
**Females (*n*)**	99	110	80	289
Age (y)	30.45 ± 5.38	50.33 ± 5.76	68.5 ± 5.82	48.55 ± 16
Body height (cm)	168.11 ± 6.95	164.91 ± 6.0	160.98 ± 5.98	164.92 ± 6.9
Body weight (kg)	67.46 ± 10.97	70.26 ± 11.59	67.8 ± 9.17	68.62 ± 10.79
**Males (*n*)**	108	91	82	281
Age (y)	30.31 ± 5.07	50.03 ± 5.69	70.3 ± 6.82	48.37 ± 17.35
Body height (cm)	179.4 ± 6.66	178.9 ± 7.2	172.59 ± 6.59	177.25 ± 7.43
Body weight (kg)	82.56 ± 12.34	87.37 ± 11.74	83.77 ± 12.95	84.47 ± 12.46
**Total (*n*)**	207	201	162	570
Age (y)	30.38 ± 5.21	50.19 ± 5.72	69.41 ± 6.34	48.46 ± 16.67
Body height (cm)	174 ± 8.83	171.24 ± 9.58	166.85 ± 8.56	171 ± 9.45
Body weight (kg)	75.34 ± 13.91	78 ± 14.42	75.89 ± 13.78	76.44 ± 14.08

Notes. Data are presented as mean ± standard deviation.

**Table 3:** Analysis of covariance (ANCOVA) summary model with bootstrap confidence intervals.

Test Position	Variables	*p*-Value	R^2^	−95 % to	95 % CI	adj. R^2^	−95 % to	95 % CI
Romberg EO	PLen	<0.001	0.39	0.33	0.46	0.38	0.32	0.46
	PLenX	<0.001	0.31	0.25	0.39	0.30	0.24	0.38
	PLenY	<0.001	0.40	0.35	0.47	0.39	0.34	0.47
	StdElA	<0.001	0.10	0.07	0.16	0.09	0.06	0.15
	BtMeanvCoF	<0.001	0.25	0.19	0.32	0.24	0.18	0.31
	BtVmeanX	<0.001	0.23	0.17	0.30	0.22	0.16	0.29
	BtVmeanY	<0.001	0.22	0.17	0.28	0.21	0.16	0.28
Semi-tandem EO	PLen	<0.001	0.43	0.36	0.52	0.43	0.36	0.51
	PLenX	<0.001	0.41	0.34	0.49	0.40	0.33	0.48
	PLenY	<0.001	0.40	0.34	0.48	0.40	0.33	0.48
	StdElA	<0.001	0.16	0.12	0.24	0.15	0.11	0.23
	BtMeanvCoF	<0.001	0.26	0.19	0.34	0.25	0.18	0.33
	BtVmeanX	<0.001	0.25	0.19	0.33	0.24	0.18	0.32
	BtVmeanY	<0.001	0.21	0.15	0.29	0.20	0.14	0.29
Tandem EO	PLen	<0.001	0.37	0.31	0.44	0.36	0.30	0.43
	PLenX	<0.001	0.40	0.34	0.47	0.39	0.33	0.46
	PLenY	<0.001	0.31	0.27	0.39	0.32	0.26	0.38
	StdElA	<0.001	0.14	0.10	0.22	0.13	0.09	0.21
	BtMeanvCoF	<0.001	0.33	0.28	0.40	0.32	0.27	0.39
	BtVmeanX	<0.001	0.39	0.34	0.46	0.38	0.33	0.45
	BtVmeanY	<0.001	0.24	0.19	0.31	0.23	0.18	0.30
One leg right EO	PLen	<0.001	0.46	0.38	0.58	0.45	0.37	0.57
	PLenX	<0.001	0.46	0.39	0.56	0.46	0.39	0.55
	PLenY	<0.001	0.43	0.34	0.55	0.42	0.33	0.54
	StdElA	<0.001	0.49	0.44	0.56	0.49	0.43	0.56
	BtMeanvCoF	<0.001	0.28	0.22	0.41	0.27	0.21	0.40
	BtVmeanX	<0.001	0.28	0.23	0.39	0.27	0.22	0.38
	BtVmeanY	<0.001	0.25	0.19	0.38	0.24	0.18	0.37
One leg left EO	PLen	<0.001	0.43	0.36	0.54	0.42	0.35	0.54
	PLenX	<0.001	0.42	0.35	0.51	0.41	0.34	0.50
	PLenY	<0.001	0.42	0.34	0.54	0.41	0.33	0.53
	StdElA	<0.001	0.45	0.40	0.53	0.45	0.39	0.52
	BtMeanvCoF	<0.001	0.25	0.20	0.36	0.24	0.19	0.36
	BtVmeanX	<0.001	0.23	0.19	0.32	0.22	0.18	0.32
	BtVmeanY	<0.001	0.26	0.21	0.38	0.25	0.20	0.37

Notes. CI: confidence interval; EO: eyes open. Statistical model includes the COP parameter as dependent variable, age group and sex as fixed factors, and body height and weight as covariates. Bootstrap CIs are based on 10,000 bootstrap samples. For abbreviations see Table 1.

## Supplementary Materials

The following are available online at https://www.mdpi.com/2411-5142/5/1/1/s1.
