# Supplementary material for: Balance Performance across the Lifespan Assessed by the Leonardo Mechanograph®: A Cross-Sectional Study
Source: J Funct Morphol Kinesiol. 2019 Dec 19;5(1):1. doi: 10.3390/jfmk5010001 (PMC7739225; doi:10.3390/jfmk5010001)
Supplement: Supplementary file 1 [file jfmk-05-00001-s001.zip › Supplement Figure S1-S4.docx]

**Figure S1-S4:** Scatterplots showing the relationship between age and each COP parameter (log-transformed)


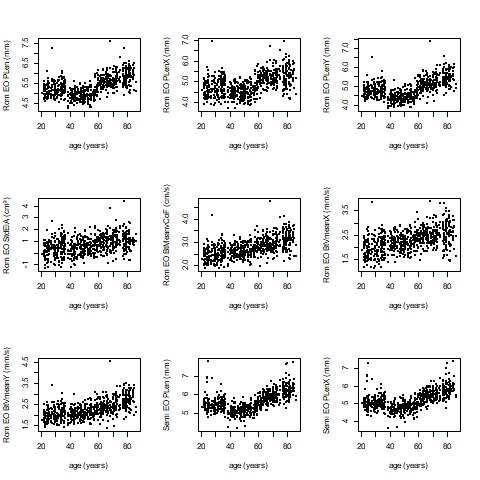
S1)


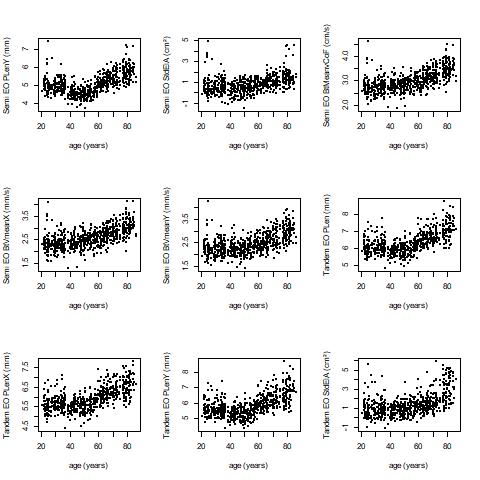
S2)


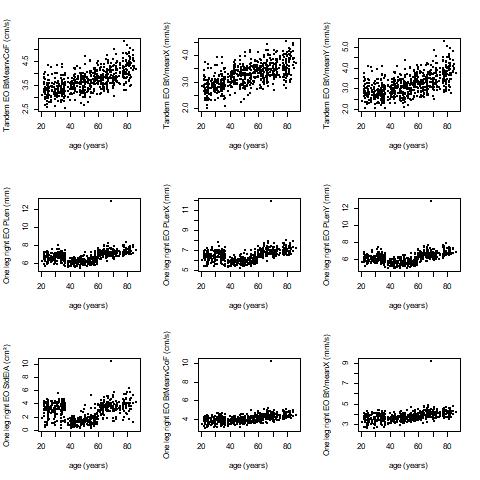
S3)


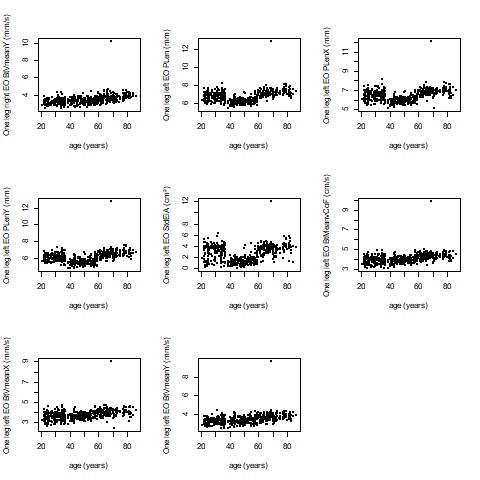
S4)
